# Confounding factors of ultrafiltration and protein analysis in extracellular vesicle research

**DOI:** 10.1038/s41598-017-02599-y

**Published:** 2017-06-02

**Authors:** Glenn Vergauwen, Bert Dhondt, Jan Van Deun, Eva De Smedt, Geert Berx, Evy Timmerman, Kris Gevaert, Ilkka Miinalainen, Véronique Cocquyt, Geert Braems, Rudy Van den Broecke, Hannelore Denys, Olivier De Wever, An Hendrix

**Affiliations:** 1 Laboratory of Experimental Cancer Research, Department of Radiation Oncology and Experimental Cancer Research, Ghent University, Ghent, Belgium; 2 VIB Medical Biotechnology Center, VIB, Ghent University, A. Baertsoenkaai 3, Ghent, Belgium; 3 Department of Biochemistry, Ghent University, A. Baertsoenkaai 3, Ghent, Belgium; 4 Department of Medical Oncology, Ghent University Hospital, Ghent, Belgium; 5 Department of Gynaecology, Ghent University Hospital, Ghent, Belgium; 6 Department of Urology, Ghent University Hospital, Ghent, Belgium; 7 Molecular and Cellular Oncology Lab, Department for Molecular Biomedical Research, Ghent, Belgium; 8 Biocenter Oulu, Department of Pathology, Oulu University Hospital, University of Oulu, Oulu, Finland; 9 Cancer Research Institute Ghent, Ghent, Belgium

## Abstract

Identification and validation of extracellular vesicle (EV)-associated biomarkers requires robust isolation and characterization protocols. We assessed the impact of some commonly implemented pre-analytical, analytical and post-analytical variables in EV research. Centrifugal filters with different membrane types and pore sizes are used to reduce large volume biofluids prior to EV isolation or to concentrate EVs. We compared five commonly reported filters for their efficiency when using plasma, urine and EV-spiked PBS. Regenerated cellulose membranes with pore size of 10 kDa recovered EVs the most efficient. Less than 40% recovery was achieved with other filters. Next, we analyzed the effect of the type of protein assays to measure EV protein in colorimetric and fluorometric kits. The fluorometric assay Qubit measured low concentration EV and BSA samples the most accurately with the lowest variation among technical and biological replicates. Lastly, we quantified Optiprep remnants in EV samples from density gradient ultracentrifugation and demonstrate that size-exclusion chromatography efficiently removes Optiprep from EVs. In conclusion, choice of centrifugal filters and protein assays confound EV analysis and should be carefully considered to increase efficiency towards biomarker discovery. SEC-based removal of Optiprep remnants from EVs can be considered for downstream applications.

## Introduction

Extracellular vesicles (EVs), nanometer-sized vesicles secreted by different cell types, gained more interest over the last decade as important key players in intercellular communication^1^ and are a promising source for new biomarkers in cancer^2–4^. Despite extensive research concerning EVs in multiple body fluids (plasma, serum and urine among others), none of the identified biomarkers are yet clinically implemented. This can partially be attributed to inter- and even intra-laboratory variations in analytical variables that hamper reproducibility. Multiple efforts have been made to standardize EV research^5–10^. Identified EV-related functions and compositions vary depending on the implemented isolation method^11, 12^. Different anticoagulants prevent with variable efficiency *in vitro* activation of cells in blood collection tubes leading to vesiculation^7, 13^.

Centrifugal filters are implemented in 20% of EV isolation protocols described in the literature^14^ (Supplementary Fig. 1). They are available with different membrane types and multiple pore sizes. The most frequently used membrane type is regenerated cellulose with a pore size of 100 kDa. Respectively 15% and 19% of studies do not specify membrane type and pore size of implemented centrifugal filters. Centrifugal filters are typically added to the EV isolation protocol to reduce large volume biofluids prior to EV isolation^15^ or to concentrate isolated EVs, for example after size-exclusion chromatography^16–18^. In addition, ultrafiltration using centrifugal filters is sometimes implemented to isolate EVs from biofluids^19, 20^.

Quantitative EV treatments in cell culture or animal models are often expressed as µg protein or number of particles. The influence of choice of particle quantification method (eg. nanoparticle tracking analysis, tunable resistive pulse sensing, high-resolution flowcytometry) to assess EV numbers have been reported^21, 22^. Diverse buffers are known to lyse EVs with different efficiency and as such influence the estimated protein concentration^23^. Multiple protein assays are available, with BCA and Bradford being the most commonly implemented to measure EV protein concentration (Supplementary Fig. 2). 35% of EV-related publications do not report on the implemented protein assay^14^.

Optiprep is a non-ionic iso-osmotic contrast agent that is used for creating continuous density gradients^24^ and effectively isolates high purity EVs from multiple body fluids^11, 25–27^. As a result, an increasing number of research groups implement Optiprep density gradient ultracentrifugation as validation or isolation method in their experiments^14^. EV samples obtained by Optiprep density gradient had higher functional activity compared to other isolation methods^28^. Interference of Optiprep with downstream omics approaches has not been reported.

In this manuscript we evaluate the effect of ultrafiltration procedures to concentrate large volume biofluids before EV isolation or to concentrate EVs after isolation. We investigate the effect of centrifugal filters and protein assays on EV samples and downstream EV analysis. In addition, we quantify Optiprep remnants in pelleted EVs obtained by Optiprep density gradient ultracentrifugation and suggest a protocol adjustment to remove Optiprep remnants from EV samples.

## Material and Methods

### Antibodies

Following antibodies were used for immunostaining: anti-green fluorescent protein (GFP) (1:1000, MAB3580, Merck Millipore, Billerica, Massachusetts, USA), anti-Syntenin-1 (1:1000, ab133267, Abcam, Cambridge, UK), anti-Flotillin-1 (1:1000, 610820, BD Biosciences, Franklin Lakes, New Jersey, USA), anti-Ago2 (1:1000, ab32381, Abcam, Cambridge, UK), anti-Alix (1:1000, 2171 S, Cell Signaling Technology, Beverly, Massachusetts, USA), anti-CD81 (1:1000, SC-166029, Santa Cruz Biotechnology, Dallas, Texas, USA), anti-TSG101 (1:1000, SC-7964, Santa Cruz Biotechnology, Dallas, Texas, USA), anti-Tamm-Horsfall (1:1000, SC-20631,, Santa Cruz Biotechnology, Dallas, Texas, USA), anti-ApoA-1 (1:1000, SC-376818, Santa Cruz Biotechnology, Dallas, Texas, USA), anti-albumin (1:1000, an28405, Abcam, Cambridge, UK), anti-IgG (1:1000, ab181236, Abcam, Cambridge, UK), anti-PMP70 (1:250, P0497, Sigma-Aldrich, St. Louis, Missouri, USA), sheep anti-mouse horseradish peroxidase-linked antibody (1:3000, NA931V, GE Healthcare Life Sciences, Uppsala, Sweden), donkey anti-rabbit horseradish peroxidase-linked antibody (1:4000, NA934V, GE Healthcare Life Sciences, Uppsala, Sweden).

### Body fluids

Venous blood from breast cancer patients was collected using Venosafe-citrate tubes (VF-054SBCS07, Terumo Europe, Leuven, Belgium). Within 120 minutes after collection, whole blood was centrifuged for 15 min at 2,500 g and room temperature, resulting in platelet poor plasma (PPP). To obtain platelet free plasma (PFP), PPP was centrifuged for 15 min at 2,500 g and room temperature. Plasma (PFP) was stored at −80 °C until further use. First morning urine was collected from bladder cancer patients and centrifuged for 10 minutes at 1,000 g and 4 °C, followed by storage at −80 °C until further use. Informed consent from all study patients was obtained prior to sample collection. Collection of patient samples was according to Ethical Committee of Ghent University Hospital approval and in accordance to relevant guidelines.

### Cell culture

The MCF-7 cell line (ATCC, Manassas, Virginia, USA) was stably transfected with peGFP-C1 vector (Clontech, Mountain View, California, USA) containing the Rab27b-GFP fusion protein, as previously described^29^. MCF-7 Rab27b-GFP cells were cultured in Dulbecco’s Modified Eagle Medium supplemented (DMEM) with 10% fetal bovine serum, 100 U/mL penicillin, 100 µg/mL streptomycin and 1 mg/mL G418. Presence of Mycoplasma contamination was routinely tested using MycoAlert Mycoplasma Detection Kit (Lonza, Verviers, Belgium).

### Preparation of conditioned medium for EV isolation

To prepare conditioned medium (CM), 4 × 10^8^ MCF-7 Rab27b-GFP cells were washed once with DMEM, followed by two washing steps with DMEM supplemented with 0.5% EV-depleted fetal bovine serum (EDS). EDS was obtained after 18 hours ultracentrifugation at 100,000 g and 4 °C (SW55 Ti rotor, Beckman Coulter, Fullerton, California, USA), followed by 0.22 µm filtration. Flasks were incubated at 37 °C and 10% CO_2_ with 15 mL DMEM containing 0.5% EDS. After 24 h CM was collected and centrifuged for 10 min at 200 g and 4 °C. Cell counting was performed with trypan blue staining to assess cell viability (Cell Counter, Life Technologies, Carlsbad, California, USA). The supernatant was passed through a 0.45 µm cellulose acetate filter (Corning, New York, USA) and CM was concentrated at 4 °C approximately 250 times using a 10 kDa Centricon Plus-70 centrifugal unit (Merck Millipore, Billerica, Massachusetts, USA). After filtering through a 0.22 µm filter (Whatman, Dassel, Germany), concentrated conditioned medium (CCM) was used for Optiprep density gradient ultracentrifugation.

### Optiprep density gradient

Optiprep (Axis-Shield, Oslo, Norway) density gradients were prepared as previously described^11^. Briefly, a discontinuous iodixanol gradient was prepared by layering 4 mL of 40%, 4 mL of 20%, 4 mL of 10% and 3.5 mL of 5% iodixanol in a 16.8 mL open top polyallomer tube (Beckman Coulter, Fullerton, California, USA). One milliliter of CCM or phosphate-buffered saline (PBS) was placed on top of the gradient, followed by 18 h ultracentrifugation at 100,000 g and 4 °C using SW 32.1 Ti rotor (Beckman Coulter, Fullerton, California, USA). Fractions of 1 mL were collected and EV-rich fractions 8 and 9 were pooled (corresponding to a density of ~1.1 g/ml). Pooled fractions were diluted to 15 mL with PBS, followed by 3 h ultracentrifugation at 100,000 g and 4 °C using SW 32.1 Ti rotor (Beckman Coulter, Fullerton, California, USA). Resulting pellets were resuspended in 100 µL PBS and stored at −80 °C until further use. When indicated, 3 h ultracentrifugation was replaced by size-exclusion chromatography. The obtained GFP-positive EVs were used in the experiments and further on referred to as ‘EVs’. Characterization of implemented EVs can be found in Supplementary Fig. 3. EV isolation and analysis details are submitted to EV-TRACK (http://evtrack.org).

EV-TRACK ID: EV170001 (EV-METRIC 100%).

### Size-exclusion chromatography

Sepharose CL-2B (GE Healthcare, Uppsala, Sweden) was washed three times with PBS containing 0.32% trisodiumcitrate dihydrate (ChemCruz, Dallas, Texas, USA). For preparation of one column, nylon net with 20 µm pore size (NY2002500, Merck Millipore, Billerica, Massachusetts, USA) was placed on bottom of a 10 mL syringe (BD Biosciences, San Jose, California, USA), followed by stacking of 10 mL Sepharose CL-2B. On top of the SEC column, 2 mL of sample was loaded and fractions of 1 mL eluate were collected. Resulting fractions were stored at −80 °C.

### Ultrafiltration

Five different centrifugal filters were used: Amicon Ultra-2 10k (UFC201024, Merck Millipore, Billerica, Massachusetts, USA), Amicon Ultra-2 100k (UFC210024, Merck Millipore, Billerica, Massachusetts, USA), Vivaspin 2 PES 10k (VS0201, Sartorius Stedim Biotech GmbH, Goettingen, Germany), Vivaspin 2 CTA 10k (VS02V1, Sartorius Stedim Biotech GmbH, Goettingen, Germany) and Vivaspin 2 Hydrosart 10k (VS02H01, Sartorius Stedim Biotech GmbH, Goettingen, Germany). 2 mL PBS spiked with 2 × 10^10^ EVs was centrifuged at 3,000 g and 4 °C (duration: 10–20 min) using a swinging bucket rotor. Concentrated samples were retrieved by upside-down centrifugation at 1,000 g and 4 °C (duration: 2 min). Eluates were collected from the flow-through reservoir. Membrane lysates were obtained by repetitive washing of the membranes with 0.4% SDS.

### Nanoparticle tracking analysis

Aliquots of isolated EVs were used for nanoparticle tracking analysis (NTA) using the NanoSight LM10 microscope (Malvern Instruments Ltd, Malvern, UK) equipped with a 455 nm laser. For each sample, three videos of 60 seconds were recorded and analyzed with camera level 13 and detection threshold 3. Temperature was monitored during recording. Recorded videos were analyzed with NTA Software version 3.2. For optimal measurements, samples were diluted with PBS until particle concentration was within optimal concentration range of the NTA Software (3 × 10^8^–1 × 10^9^).

### Electron microscopy

Aliquots of EVs were used for transmission electron microscopy. Sample was deposited on Formvar carbon-coated, glow discharged grids. Grids were stained with uranylacetate and embedded in methylcellulose/uranylacetate. Prepared grids were examined using a Tecnai Spirit transmission electron microscope (FEI, Eindhoven, The Netherlands) and images were captured with Quemasa charge-coupled device camera (Olympus Soft Imaging Solutions GMBH, Munster, Germany).

### Western blot

All samples were dissolved in reducing sample buffer (0,5 M Tris-HCl (pH 6.8), 40% glycerol, 9,2% SDS, 3% 2-mercaptoethanol, 0.005% bromophenol blue) and boiled at 95 °C for 5 min. Proteins were separated by SDS-polyacrylamide gel electrophoresis and transferred to nitrocellulose membranes (Bio-Rad, Hercules, California, USA). After blocking the membranes, blots were incubated overnight with primary antibodies. Incubation with secondary antibodies was performed after extensive washing of the membranes in PBS containing 0,5% Tween 20. After final washing, chemiluminescence substrate (WesternBright Sirius, Advansta, Menlo Park, California, USA) was added and imaging was performed using Proxima 2850 Imager (IsoGen Life Sciences, De Meern, The Netherlands). Quantification of signal intensity was performed using ImageJ software.

### TRIFic exosome assay

Quantification of CD9 was performed using the TRIFic exosome assay (Cell Guidance Systems, Carlsbad, California, USA) according to manufacturer’s protocol. Briefly, a 96-well plate was coated with the capture CD9 antibody, followed by addition of the samples in the 96-well plate. Europium-labeled detection CD9 antibody was added afterwards. Each step extensive washing was performed. Signal enhancement solution was added and fluorescence was measured. Results were obtained using the Paradigm Detection Platform (Beckman Coulter, Fullerton, California, USA) using SoftMax Pro 6.1 software (Molecular Devices, Sunnyvale, California, USA).

### Optiprep quantification based on Lowry interference

Quantification of residual Optiprep was based on the observed interference of iodixanol with Lowry-based protein measurement. A dilution series of 0 to 10% of Optiprep in PBS was obtained as a standard curve for performing absorbance readings with DC Protein assay (Bio-Rad, Hercules, California, USA), according to manufacturer’s instructions. Pelleted Optiprep fractions 8–9 of blank gradients were used as sample for measuring absorbance. Calculations of Optiprep were made using the previouly described Optiprep dilution series as standard curve. Readings were performed on the Paradigm Detection Platform (Beckman Coulter, Fullerton, California, USA) using SoftMax Pro 6.1 software (Molecular Devices, Sunnyvale, California, USA).

### Protein quantification assays

Seven different protein assays were compared. In all assays, 5 µL of EVs (lysing condition SDS 0.2%) derived from the MCF-7 Rab27b-GFP cell line were used as sample. According to manufacturer’s instructions, the 5 µL sample was diluted with PBS to assay sample volume. The microplate protocol in assay-specific datasheet was performed using following protein assays: DC Protein assay (Bio-Rad, Hercules, California, USA), Pierce BCA protein assay (Life Technologies, Carlsbad, California, USA), Coomassie Plus Bradford assay (Life Technologies, Carlsbad, California, USA) and MicroBCA protein assay (Life Technologies, Carlsbad, California, USA). Two fluorometric assays were performed according to manufacturer’s instructions: NanoOrange (Life Technologies, Carlsbad, California, USA) and FluoroProfile (Sigma-Aldrich, St. Louis, Missouri, USA). Absorbance and fluorescence readings were obtained using the Paradigm Detection Platform (Beckman Coulter, Fullerton, California, USA) using SoftMax Pro 6.1 software (Molecular Devices, Sunnyvale, California, USA). A third fluorometric assay was used: Qubit Protein assay kit (ThermoFisher, Waltham, Massachusetts, USA), measurements were performed using the Qubit Fluorometer 3.0 (ThermoFisher, Waltham, Massachusetts, USA).

### RNA extraction and RT-qPCR

RNA was extracted using miRNeasy micro kit (Qiagen, Valencia, California, USA), according to manufacturer’s instructions. RNA was reverse transcribed to cDNA with Sensifast cDNA Synthesis Kit (GC Biotech, Alphen aan den Rijn, The Netherlands). RT-qPCR for gene expression analysis was performed using SensiFast SYBR No-Rox kit (GC Biotech, Alphen aan den Rijn, The Netherlands) on LightCycler 480 (Roche, Brussels, Belgium). The used primers can be found in Supplementary Table 1. Gene expression was assessed relatively to initial spike-in RNA amount. Analysis was performed using qBase + software 3.0 (Biogazelle, Ghent, Belgium).

### Statistical analysis

Data analysis and graphical presentations were performed using GraphPad Prism version 7.02 (Graphpad Software, San Diego, California, USA). An unpaired Student’s *t*-test was applied for determination of significant difference between conditions. P values of less than 0.05 indicated statistical significance. Data are expressed as mean ± SD.

## Results

### Impact of centrifugal filters on particle and protein recovery from biofluids

**Figure 1:**
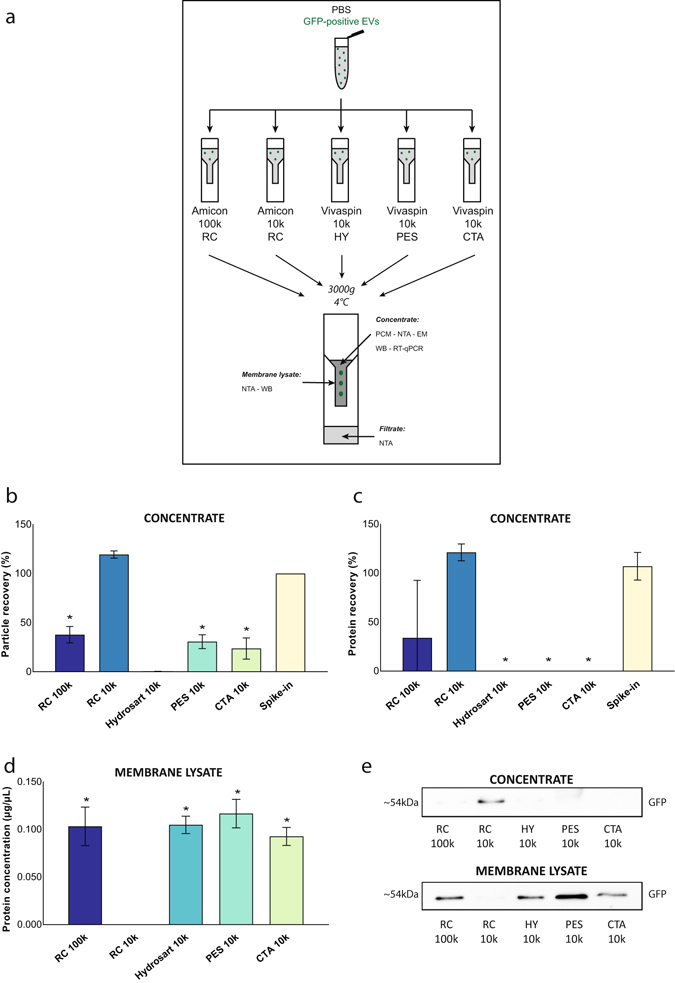
Comparative analysis of centrifugal filters for protein and particle recovery of EVs in PBS. (**a**) 2 mL PBS spiked with GFP-positive EVs was centrifuged at 3,000 g. Concentrate and filtrate were collected. Lysates of filter membranes were used for analysing retained proteins. (**b**) Particle recovery was analyzed using Nanoparticle tracking analysis (n = 3, *p < 0.0001). (**c**) Protein recovery was assessed based on protein concentration measurements (n = 3, *p < 0.0001). (**d**) Retained proteins on the filter membrane were quantified (n = 3, *p < 0.0009). (**e**) Lysates were used for Western blot immunostaining for GFP. Mean values + SD are indicated. Significant differences were calculated using Student’s t-test with RC 10k as reference. Original immunostaining results are shown in Supplementary Fig. 10. Abbreviations: PBS: phosphate buffered saline. GFP: green fluorescent protein. EV: extracellular vesicle. RC: regenerated cellulose. HY: Hydrosart. PES: polyethersulfone. CTA: cellulose triacetate. WB: Western blot. NTA: Nanoparticle tracking analysis. PCM: protein concentration measurement. RT-qPCR: real time quantitative polymerase chain reaction. EM: electron microscopy.

The efficiency of five different centrifugal filters was compared to concentrate a defined number of isolated EVs spiked in PBS (10^10^ EVs/mL): Amicon 100k RC, Amicon 10k RC, Vivaspin 10k HY, Vivaspin 10k PES and Vivaspin 10k CTA (Table 1). For each column, 2 mL sample was concentrated 20-fold (100 µL) by centrifugation at 3,000 g and 4 °C. Three end-point samples per column were analyzed: the concentrate, the filtrate and the membrane lysate (Fig. 1a). These samples were analyzed by NTA, electron microscopy, protein concentration measurement, Western blot analysis and/or RT-qPCR.

**Table 1:** Characteristics of implemented centrifugal filters.

	Membrane	Pore size
Amicon 10k RC	regenerated cellulose	10 kDa
Amicon 100k RC	regenerated cellulose	100 kDa
Vivaspin 10k HY	Hydrosart	10 kDa
Vivaspin 10k PES	polyethersulfone	10 kDa
Vivaspin 10k CTA	cellulose triacetate	10 kDa

Amicon 10k RC generated the highest particle and protein recovery ([particle or protein concentration after centrifugation/particle or protein concentration before centrifugation] * 100) of EVs in the concentrate (Fig. 1b,c). Amicon 100k RC recovered significantly less EVs as compared to Amicon 10k RC. Other centrifugal membranes (Vivaspin 10k HY, Vivaspin 10k PES and Vivaspin 10k CTA) did not efficiently recover EVs in the concentrate with up to 80% reduction in particle concentration and protein concentration measurements below detection threshold. Size distribution profiles and electron microscopy images of concentrated EV samples are summarized in Supplementary Fig. 4. NTA measurements of filtrates for all centrifugal filters were below detection threshold, excluding passage of EVs through the membrane pores (data not shown).

Protein concentration was above detection threshold for all lysates of the membranes of the centrifugal filters, except for the Amicon 10k RC where no protein was detected (Fig. 1d). Western blot analysis for GFP of both the concentrate and the membrane lysate showed that EVs were retained in the concentrate using the Amicon 10k RC, with the absence of EVs in the membrane lysate. By contrast, for all the other centrifugal filters, the majority of EVs are recovered in the membrane lysate (Fig. 1e).

**Figure 2:**
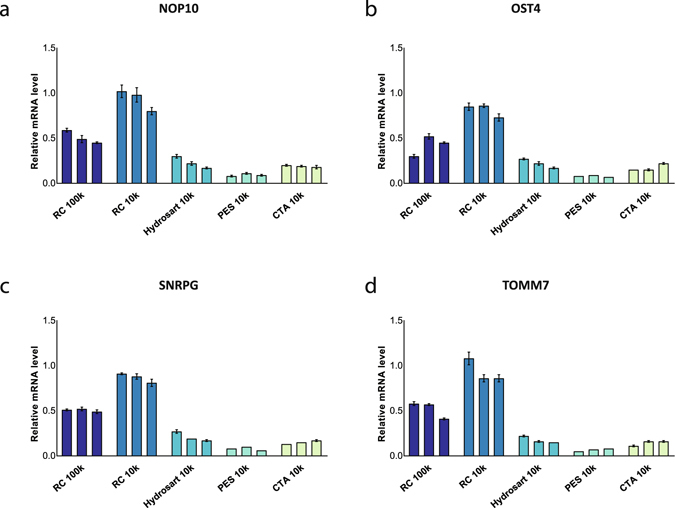
Comparative analysis of centrifugal filters for EV-associated RNA recovery in PBS. Four genes present in EVs derived from MCF-7 Rab27b-GFP cell line were assessed using RT-qPCR. Results are expressed relatively to spike-in EV RNA level for (**a**) NOP10, (**b**) OST4, (**c**) SNRPG and (**d**) TOMM7. Triplicate experiments are indicated by three bars per condition. Mean values + SEM are indicated (n = 3). Abbreviations: PBS: phosphate buffered saline. EV: extracellular vesicle. RC: regenerated cellulose. HY: Hydrosart. PES: polyethersulfone. CTA: cellulose triacetate. RT-qPCR: real time quantitative polymerase chain reaction.

Influence of centrifugal filter on RNA analysis was studied by RT-qPCR for all different centrifugal filters. We selected four genes (NOP10, OST4, SNRPG, TOMM7) known to be present in EVs from MCF-7 breast cancer cells overexpressing Rab27b-GFP^11^. For all four genes, identical observations were made as with particle and protein analysis, where Amicon 10k RC showed the highest RNA signal compared to the other centrifugal filters (Fig. 2).

**Figure 3:**
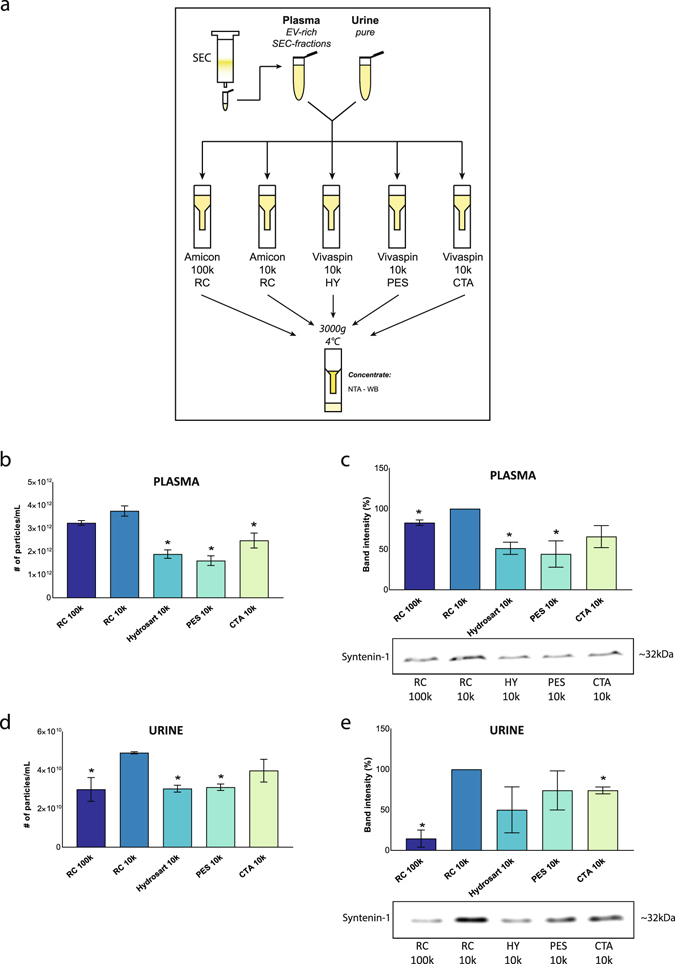
Comparative analysis of centrifugal filters for protein and particle recovery from plasma and urine. (**a**) Blood plasma was loaded on SEC column and EV-rich fractions were concentrated using five different centrifugal filters. Urine was concentrated using the same type of filters. (**b**) Nanoparticle Tracking Analysis (n = 2, *p < 0.05) and (**c**) Western blot analysis for syntenin-1 was performed on concentrated EV-rich SEC-fractions from plasma (n = 2, *p < 0.04). Western blot analysis was quantified using RC 10k as reference. (**d**) Concentrated urine was analyzed by Nanoparticle tracking analysis (n = 2, *p < 0.05) and (**e**) Western blot analysis for syntenin-1 (n = 2, *p < 0.02). Western blot analysis was quantified using RC 10k as reference in (**c**,**e**). Mean values + SD are indicated. Significant differences were calculated using Student’s t-test with RC 10k as reference. Original immunostaining results are shown in Supplementary Fig. 10. Abbreviations: SEC: size-exclusion chromatography. EV: extracellular vesicle. RC: regenerated cellulose. HY: Hydrosart. PES: polyethersulfone. CTA: cellulose triacetate. WB: Western blot. NTA: Nanoparticle tracking analysis. PCM: protein concentration measurement.

Next, the centrifugal filters were applied to two commonly used body fluids: plasma and urine. The concentrate, retrieved after centrifugation at 3,000 and 4 °C (duration 20–40 min), was analyzed by NTA, protein concentration measurement and Western bot analysis (Fig. 3a). Since plasma is a protein-rich body fluid, size-exclusion chromatography was performed to obtain a protein-poor, EV-enriched fraction which was subsequently concentrated by ultrafiltration (Supplementary Fig. 5). The highest number of particles was obtained in the concentrate of Amicon 10k RC. Implementing Amicon 100k RC reduced particle recovery. However, the loss of particles was drastically higher in the concentrate from the other membranes (Fig. 3b). Analogous results were observed by measuring protein concentration. The highest protein recovery in the concentrate was obtained by using Amicon 10k RC. These results were reflected on Western blot analysis for syntenin-1 (Fig. 3c). Also for urine, NTA showed the highest concentration of particles in the concentrate obtained by Amicon 10k RC (Fig. 3d), which was confirmed by protein recovery and syntenin-1 quantification (Fig. 3e). Western blot analysis of the membrane lysates identified soluble proteins and lipoproteins on all membranes, suggesting nonspecific binding of these proteins to the membranes (Supplementary Fig. 6).

In conclusion, centrifugal filters with a regenerated cellulose membrane with a pore size of 10 kDa are required to concentrate EVs without significant loss.

### Impact of protein assays on the quantification of EV protein

**Figure 4:**
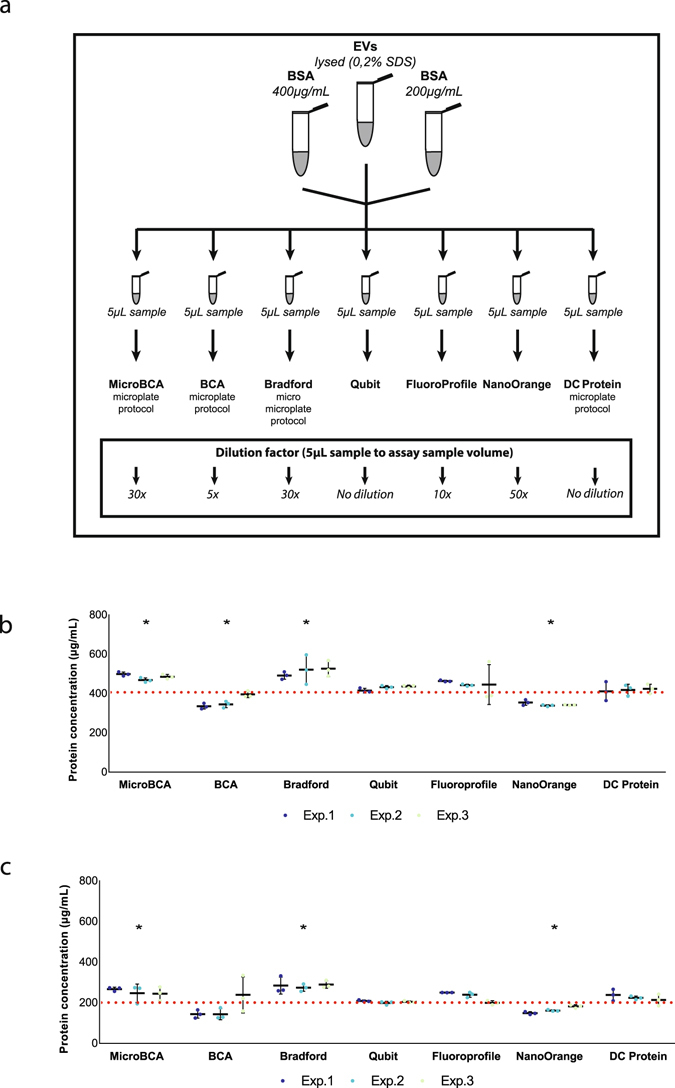
Comparison of the performance of different protein assays to measure protein concentration of BSA and EV samples. (**a**) Seven different protein assays (four colorimetric, three fluorometric) were compared using three different samples: BSA 400 µg/mL, BSA 200 µg/mL and EV sample. Identical sample volume was used in all assays and proper dilution to working sample volume was implemented. (**b,c**) All protein assays were performed on two known BSA concentrations: (**b**) 400 µg/mL (n = 3, *p < 0.003) and (**c**) 200 µg/mL (n = 3, *p < 0.05). Red dotted line indicates known BSA concentration. Mean values + SD are indicated. Significant differences were calculated using Student’s t-test with Qubit as reference. Abbreviations: EV: extracellular vesicle. BSA: bovine serum albumin. Exp.: Experiment.

The performance of four colorimetric assays (DC Protein, BCA, MicroBCA, Bradford) and three fluorometric assays (Qubit, NanoOrange, FluoroProfile) was compared on three different sample types: a lysed EV sample and two BSA solutions with known concentrations (400 µg/mL and 200 µg/mL). Supplementary Table 2 indicates the main characteristics and differences between the implemented protein assays. In compliance with the manufacturer’s guidelines, a corresponding dilution was implemented to obtain the optimal sample volume for each individual assay (Fig. 4a). For all protein assays the same starting volume of 5 µL EVs was used, corresponding to 2 × 10^10^ particles. BSA, a protein commonly used as reference or internal standard in protein quantification methods, was used to assess variation in protein measurements between different protein assays. Measuring both low BSA concentrations (400 µg/mL and 200 µg/mL), we observed variation throughout the different protein assays, with least variance for the most accurate measurements by Qubit and DC Protein (Fig. 4b,c). Interference of Folin’s reagent, a main component of DC Protein and all other Lowry-based protein assays, with iodixanol has previously been described^24^ and thereby allows to quantify Optiprep remnants in EV pellets. We prepared a standard curve of Optiprep using the DC Protein assay (Supplementary Fig. 7a). The Optiprep concentration of pelleted EV density fractions from a blank Optiprep gradient (PBS as sample) was estimated at 1.5–2.5% (Supplementary Fig. 7b,c). Comparison of all seven protein assays for interference of Optiprep using a 3% Optiprep-containing PBS sample indicated major interference for the DC Protein assay (Supplementary Fig. 8). Due to this interference, further comparison of protein assays on EV samples were performed for all protein assays except the Lowry-based DC Protein assay.

**Figure 5:**
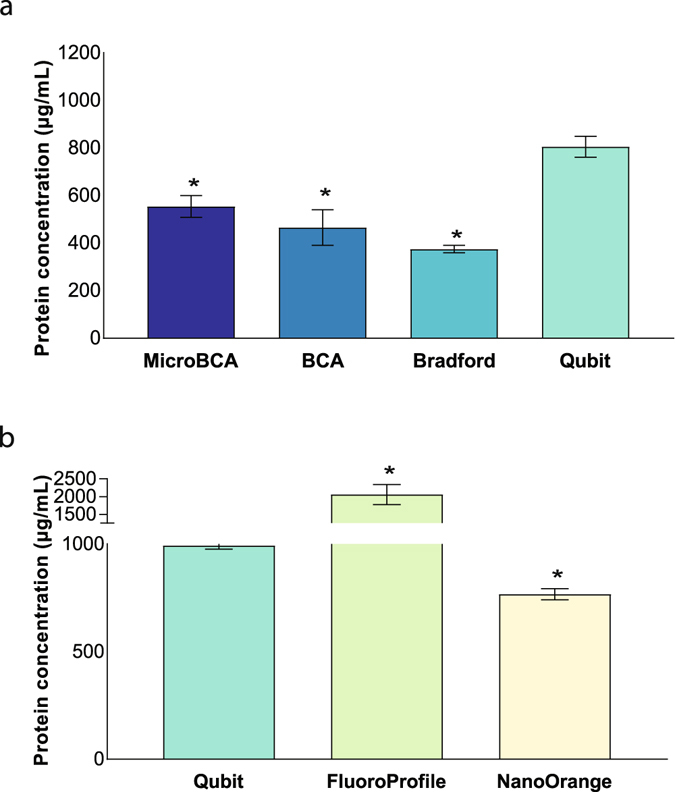
Performance of different protein assays to measure protein concentration of EV samples. (**a**) Protein quantification for one EV sample using three colorimetric assays (MicroBCA, BCA, Bradford) was performed opposed to one fluorometric assay (Qubit) (n = 3, *p < 0.003). (**b**) All fluorometric assays (Qubit, FluoroProfile, NanoOrange) were tested on another EV-sample (n = 3, *p < 0.005). Mean values + SD are indicated. Significant differences were calculated using Student’s t-test with Qubit as reference. Abbreviations: EV: extracellular vesicle. BSA: bovine serum albumin.

Differences in protein concentration measurement were observed dependent upon the implemented protein assay (Fig. 5a,b). The Qubit assay obtained a respectively 1.5-fold and 2-fold higher protein concentration measurement as compared to MicroBCA and Bradford. The difference in obtained results was not due to interference of Optiprep, as shown in Fig. 5c. Protein concentration measurements of EV lysates before and after one freeze-thaw cycle revealed no significant difference within any assay (Supplementary Fig. 9).

Taken together, Qubit assay is able to accurately measure low BSA concentration with least variance compared to other protein assay kits. This low variance was also confirmed for EV samples, suggesting it to be a suitable assay for protein quantification.

### Removal of Optiprep remnants in EV samples

**Figure 6:**
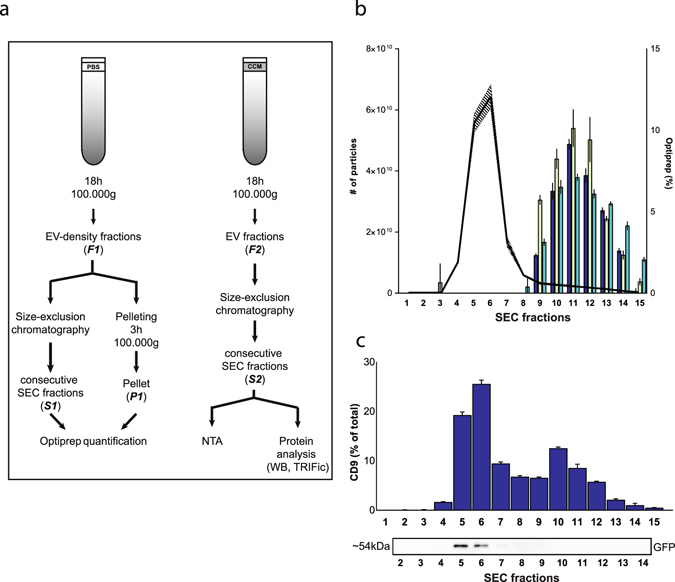
The implementation of size-exclusion chromatography to remove Optiprep remnants from EV samples. (**a**) Optiprep density gradients were loaded with PBS to obtain blank EV-density fractions (F1) and with CCM to obtain EV-containing fractions (F2). EVs were retrieved by pelleting at 100,000 g (P1) or by size-exclusion chromatography (S1). Quantity of Optiprep in S1 and P1 was calculated using DC Protein assay. SEC was performed on F2 and consecutive SEC fractions (S2) were analyzed by NTA and protein analysis for GFP and CD9. (**b**) Comparative graph of NTA from S2 (connected line, marked area = SD) and Optiprep quantification of S1 (three differently colored bars representing three independent gradients, SD indicated). (**c**) Protein analysis of S2 by Western blot analysis for GFP and CD9 quantification by TRIFic CD9 assay. Original immunostaining results are shown in Supplementary Fig. 10. Abbreviations: PBS: phosphate buffered saline. CCM: concentrated conditioned medium. GFP: green fluorescent protein. EV: extracellular vesicle. WB: Western blot. NTA: Nanoparticle tracking analysis. SEC: size-exclusion chromatography.

To remove the previously mentioned Optiprep remnants from EV samples, we investigated the use of size-exclusion chromatography as an alternative to 100,000 g pelleting for 3 hours (Fig. 6a). Optiprep quantification in SEC fractions using the DC Protein assay was performed and indicated that Optiprep elution starts from SEC fraction 8–9 onwards (Fig. 6b). The majority of EVs from EV-containing Optiprep density fractions prepared from cell culture supernatant elute in SEC fractions 4–7 (Fig. 6b). Western blot immunostaining for GFP and CD9 quantification using an ELISA assay confirmed that the particles eluted in SEC fractions 4–7 were CD9 and GFP positive (Fig. 6c).

In conclusion, removal of Optiprep remnants in EV samples can be obtained by size-exclusion chromatography as alternative of EV pelleting by ultracentrifugation at 100,000 g.

## Discussion

Important yet undervalued sources of variability that impair reproducibility of EV results are identified. Ultrafiltration or centrifugal concentration are important steps in EV isolation protocols. Several companies offer centrifugal filters with different membrane types. The choice of centrifugal filter determines the recovery of EVs from samples. In addition, EV research papers do not adequately report the choice of ultrafiltration filter^14^. Both elements may severely impact the reproducibility of isolation protocols. The variability in recovery is due to different binding capacities of EVs to various membrane filters. Nonspecific binding of compounds to centrifugal membranes has already been described^30^, yet for EV research this information was lacking. Treating the membranes with detergents reduced nonspecific binding of compounds to the membrane^31^ but resulting remnant detergent is an obvious obstacle for EV isolation. Regenerated cellulose was shown to be the least adhesive membrane for EVs and thereby the most optimal membrane for handling EV samples. Moreover, not only type of filter membrane appeared important, but also the pore size influenced recovery. Regenerated cellulose membranes with a 100 kDa pore size resulted in adhesion of EVs on the membrane, while this effect was not observed in a 10 kDa pore size membrane of regenerated cellulose. The differences between the centrifugal filters was lower when applied to plasma and urine as compared to the *in vitro* set-up with EVs spiked into PBS. This is likely due to the presence of soluble proteins and lipoproteins in body fluids that adhere to the membranes and thereby partially prevent EVs from binding to the membranes. However, despite the competitive nonspecific binding of proteins, still a substantial amount of EVs adhere to the filter membranes. Our data suggest to test different centrifugal filters for different sample types prior to their implementation in ultrafiltration or concentration and to correctly report their use in materials and methods sections for optimal reproducibility of experiments between research groups.

Quantitative experiments (eg. recovery descriptions, functional assays *in vitro* and *in vivo*) are not only confounded by particle number^21, 22^, but also by protein quantification dependent on the type of protein assay kit used. Although within-kit measurements showed high repeatability, between-kit measurements resulted in major quantitative differences. If the type of protein assay is not reported in the materials and methods section, reproducibility among research groups is hampered. Comparison studies of protein assays have been described previously^32–34^, yet this comparison for EV samples is still lacking. *Kirazov et al*. encountered underestimation of protein when membrane-containing samples were measured using Bradford assay^33^. In agreement, the Bradford assay underestimated EV protein concentrations. Measuring protein content of outer membrane vesicles (OMVs) from bacteria had less variance using Lowry-based assay than using Bradford, again resulting in lower measurements when using Bradford assay^32^. Comparison of six different protein assay kits indicated highly different results on analysis of EV samples, while this difference was present but less pronounced using BSA standards (200–400 µg/mL). The fluorometric assay Qubit showed the least variance between different experiments and was able to obtain results the closest to the known BSA concentrations. Given this accurate measurement, combined with the absence of multiple manipulations in the protocol or sample dilution, Qubit is a reproducible and easy method for protein quantification of EV samples.

Optiprep remnants in isolated EV pellets are between 1.5–3%. The importance of residual isolation matrix in obtained EVs has been decribed, showing Optiprep-isolated EVs to be more functionally active than those from eg. precipitation techniques^28^. This higher functional activity was attributed to the presence of residual matrix in the precipitation techniques. However, the influence of residual Optiprep was not investigated. Optiprep was removed from EV samples by SEC, as was already described for the isolation of adenoviral vectors^35^. Using DC Protein assay, based on interference of Optiprep with this Lowry-based protein assay, the removal of Optiprep from EVs was confirmed. The classic pelleting step at 100,000 g for 3 hours is replaced by a SEC step. SEC preserves functional and biological activities of EVs^36^ and is, in contrast to ultracentrifugation, not known for inducing aggregation of EVs^37^. Further knowledge of the benefit of Optiprep removal from EV samples still needs to be determined, especially regarding functional activity of isolated EVs and the impact on downstream omics approaches.

In conclusion, minor protocol parameters, such as type of centrifugal filter and type of protein assay, can have a major impact on data from EV experiments. In addition, when implementing Optiprep density gradient centrifugation, removal of Optiprep may be considered for downstream applications. It is crucial to report each protocol step properly to enhance reproducibility among research groups. Especially when working with a low amount of EVs, appropriate choices should be made to ensure optimal EV recovery, quantitative estimations and protein or RNA identification.

## Electronic supplementary material

Supplementary Information

## References

[1] Simons M, Raposo G (2009). Exosomes - vesicular carriers for intercellular communication. Curr. Opin. Cell Biol..

[2] Melo SA (2015). Glypican-1 identifies cancer exosomes and detects early pancreatic cancer. Nature.

[3] Taylor DD, Gercel-Taylor C (2008). MicroRNA signatures of tumor-derived exosomes as diagnostic biomarkers of ovarian cancer. Gynecol. Oncol..

[4] Ciardiello C (2016). Focus on Extracellular Vesicles: New Frontiers of Cell-to-Cell Communication in Cancer. Int. J. Mol. Sci..

[5] Lötvall J (2014). Minimal experimental requirements for definition of extracellular vesicles and their functions: a position statement from the International Society for Extracellular Vesicles. J. Extracell. Vesiclesvesicles.

[6] Witwer KW (2013). Standardization of sample collection, isolation and analysis methods in extracellular vesicle research. J. Extracell. vesicles.

[7] Lacroix R (2012). Impact of pre-analytical parameters on the measurement of circulating microparticles: towards standardization of protocol. J. Thromb. Haemost.

[8] Yuana Y, Böing A, Grootemaat A, Hau C, Cizmar E (2015). Handling and storage of human body fluids for analysis of extracellular vesicles. J. Extracell. Vesicles.

[9] Yuana Y, Bertina RM, Osanto S (2011). Pre-analytical and analytical issues in the analysis of blood microparticles. Thromb. Haemost..

[10] van Deun J (2017). EV-TRACK: transparent reporting and centralizing knowledge in extracellular vesicle research. Nat. Methods.

[11] van Deun J (2014). The impact of disparate isolation methods for extracellular vesicles on downstream RNA profiling. J. Extracell. Vesicles.

[12] Willms E (2016). Cells release subpopulations of exosomes with distinct molecular and biological properties. Sci. Rep.

[13] György B (2014). Improved circulating microparticle analysis in acid-citrate dextrose (ACD) anticoagulant tube. Thromb. Res..

[14] van Deun, J. *et al*. EV-TRACK: Transparent Reporting and Centralizing Knowledge in EV research. at www.evtrack.org. 10.1038/nmeth.4185 28245209

[15] Gardiner, C. *et al*. *Techniques used for the isolation and characterization of extracellular vesicles: results of a worldwide survey* **1**, 1–6 (2016). 10.3402/jev.v5.32945 PMC5090131 27802845

[16] de Menezes-Neto, A. *et al*. Size-exclusion chromatography as a stand-alone methodology identifies novel markers in mass spectrometry analyses of plasma-derived vesicles from healthy individuals. *J*. *Extracell*. *Vesicles* **4** (2015). 10.3402/jev.v4.27378 PMC4495624 26154623

[17] Welton, J. L., Webber, J. P., Botos, L.-A., Jones, M. & Clayton, A. Ready-made chromatography columns for extracellular vesicle isolation from plasma. *J*. *Extracell*. *Vesicles* **4** (2015). 10.3402/jev.v4.27269 PMC4376847 25819214

[18] Boïng, A. N. *et al*. Single-step isolation of extracellular vesicles by size-exclusion chromatography. *J*. *Extracell*. *Vesicles* **3** (2014). 10.3402/jev.v3.23430 PMC4159761 25279113

[19] Goldie BJ (2014). Activity-associated miRNA are packaged in Map1b-enriched exosomes released from depolarized neurons. Nucleic Acids Res.

[20] Merchant ML (2010). Microfiltration isolation of human urinary exosomes for characterization by MS. Proteomics - Clin. Appl.

[21] Akers JC (2016). Comparative analysis of technologies for quantifying extracellular vesicles (EVs) in clinical cerebrospinal fluids (CSF). PLoS One.

[22] Maas SLN (2015). Possibilities and limitations of current technologies for quantification of biological extracellular vesicles and synthetic mimics. J. Control. Release.

[23] Osteikoetxea X (2015). Differential detergent sensitivity of extracellular vesicle subpopulations. Org. Biomol. Chem..

[24] Ford T, Graham J, Rickwood D (1994). Iodixanol: A nonionic iso-osmotic centrifugation medium for the formation of self-generated gradients. Anal. Biochem..

[25] Shiba K (2016). Isolation of human salivary extracellular vesicles by iodixanol density gradient ultracentrifugation and their characterizations. J. Extracell. Vesicles.

[26] Kalra H (2013). Comparative proteomics evaluation of plasma exosome isolation techniques and assessment of the stability of exosomes in normal human blood plasma. Proteomics.

[27] Raimondo F (2013). Differential protein profiling of renal cell carcinoma urinary exosomes. Mol. Biosyst..

[28] Paolini L (2016). Residual matrix from different separation techniques impacts exosome biological activity. Sci. Rep.

[29] Hendrix A (2010). Effect of the secretory small GTPase Rab27B on breast cancer growth, invasion, and metastasis. J. Natl. Cancer Inst..

[30] Kratzer A, Kees F, Dorn C (2016). Unbound fraction of fluconazole and linezolid in human plasma as determined by ultrafiltration: Impact of membrane type. J. Chromatogr. B.

[31] Lee KJ (2003). Modulation of nonspecific binding in ultrafiltration protein binding studies. Pharm. Res..

[32] Rossi O (2014). Comparison of Colorimetric Assays with Quantitative Amino Acid Analysis for Protein Quantification of Generalized Modules for Membrane Antigens (GMMA). Mol. Biotechnol..

[33] Kirazov LP, Venkov LG, Kirazov EP (1993). Comparison of the Lowry and the Bradford protein assays as applied for protein estimation of membrane-containing fractions. Anal. Biochem..

[34] Okutucu B, Dınçer A, Habib Ö, Zıhnıoglu F (2007). Comparison of five methods for determination of total plasma protein concentration. J. Biochem. Biophys. Methods.

[35] Peng, H. H. *et al*. A rapid and efficient method for purification of recombinant adenovirus with RGD-modified fibers **354**, 140–147 (2007). 10.1016/j.ab.2006.04.032 PMC1475777 16707084

[36] Nordin JZ (2015). Ultrafiltration with size-exclusion liquid chromatography for high yield isolation of extracellular vesicles preserving intact biophysical and functional properties. Nanomedicine Nanotechnology, Biol. Med.

[37] Linares R, Tan S, Gounou C, Arraud N, Brisson AR (2015). High-speed centrifugation induces aggregation of extracellular vesicles. J. Extracell. vesicles.

