# Supplementary material for: Confounding factors of ultrafiltration and protein analysis in extracellular vesicle research
Source: Sci Rep. 2017 Jun 2;7:2704. doi: 10.1038/s41598-017-02599-y (PMC5457435; doi:10.1038/s41598-017-02599-y)
Supplement: Supplementary file 1 — Supplementary Information [file 41598_2017_2599_MOESM1_ESM.pdf]

Confounding factors of ultrafiltration and protein analysis in extracellular vesicle research

Glenn Vergauwen[1,5,9], Bert Dhondt[1,6,9], Jan Van Deun[1,9], Eva De Smedt[7,9], Geert Berx[7,9], Evy Timmerman[2,3], Kris Gevaert[2,3], Ilkka Miinalainen[8], Véronique Cocquyt[4,9], Geert Braems[5,9], Rudy Van den Broecke[5,9], Hannelore Denys[4,9], Olivier De Wever[1,9], An Hendrix[1,9].

Affiliations

[1] Laboratory of Experimental Cancer Research, Department of Radiation Oncology and Experimental Cancer Research, Ghent University, Ghent, Belgium.
[2] VIB Medical Biotechnology Center, VIB, Ghent University, A. Baertsoenkaai 3, Ghent, Belgium.
[3] Department of Biochemistry, Ghent University, A. Baertsoenkaai 3, Ghent, Belgium.
[4] Department of Medical Oncology, Ghent University Hospital, Ghent, Belgium.
[5] Department of Gynaecology, Ghent University Hospital, Ghent, Belgium.
[6] Department of Urology, Ghent University Hospital, Ghent, Belgium.
[7] Molecular and Cellular Oncology Lab, Department for Molecular Biomedical Research, Ghent, Belgium.
[8] Biocenter Oulu, Department of Pathology, Oulu University Hospital, University of Oulu, Oulu, Finland.
[9] Cancer Research Institute Ghent, Ghent, Belgium

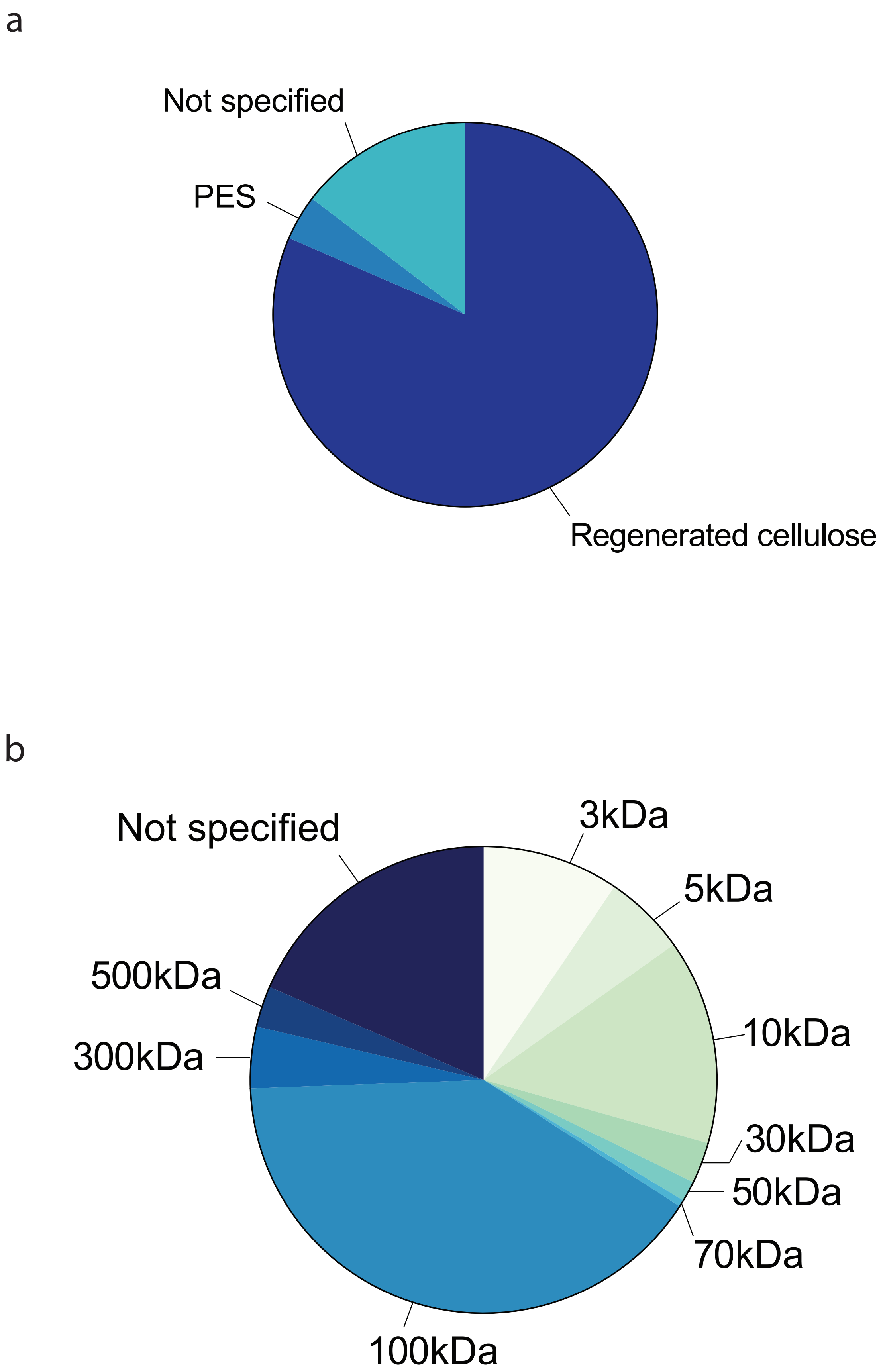

Supplementary figure 1: Heterogeneity in the use and reporting of centrifugal filters
The EV-TRACK knowledgebase was used for visualizing heterogeneity in the use of centrifugal filters in EV-related research papers. (a) Reported membrane types. (b) Reported pore sizes.

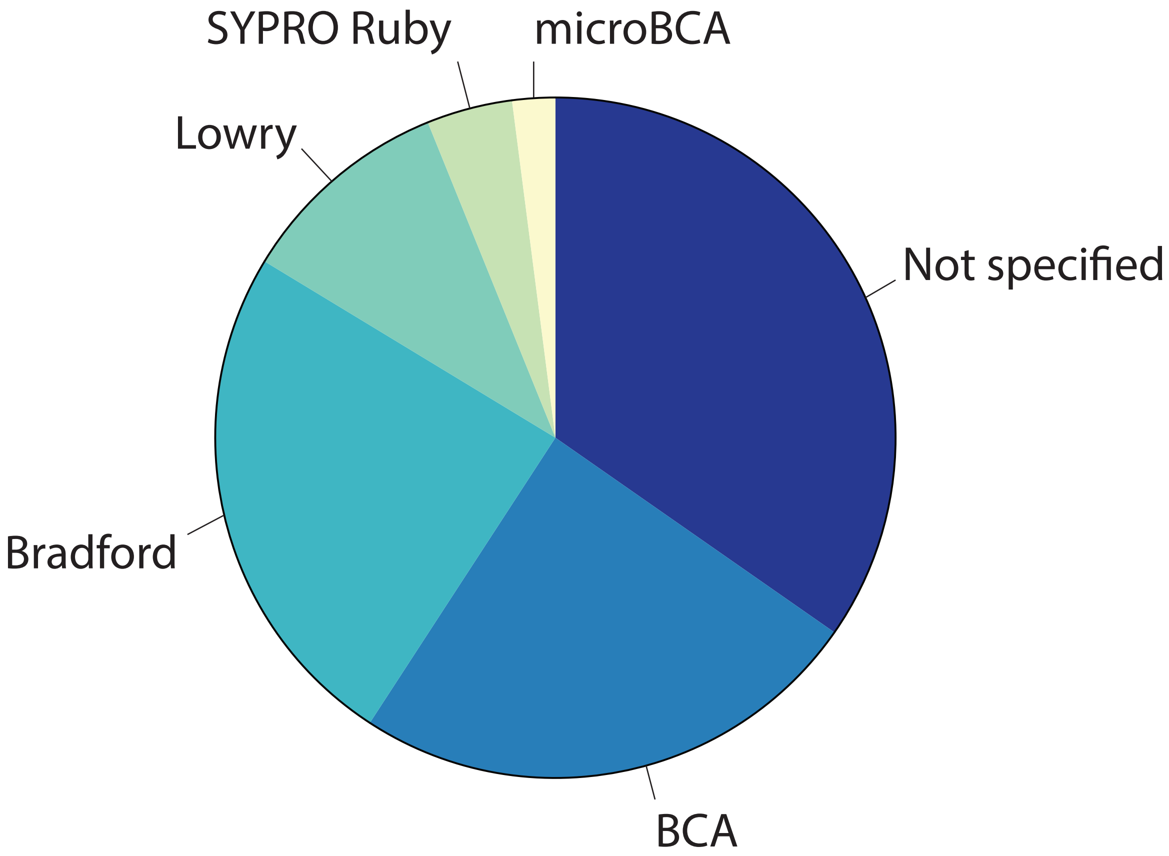

Supplementary figure 2: Multiple protein assays are implemented to measure EV protein concentration
The EV-TRACK knowledgebase was used for visualizing heterogeneity in EV-related research papers concerning the use of protein assays in papers that mention protein quantity of EV samples.

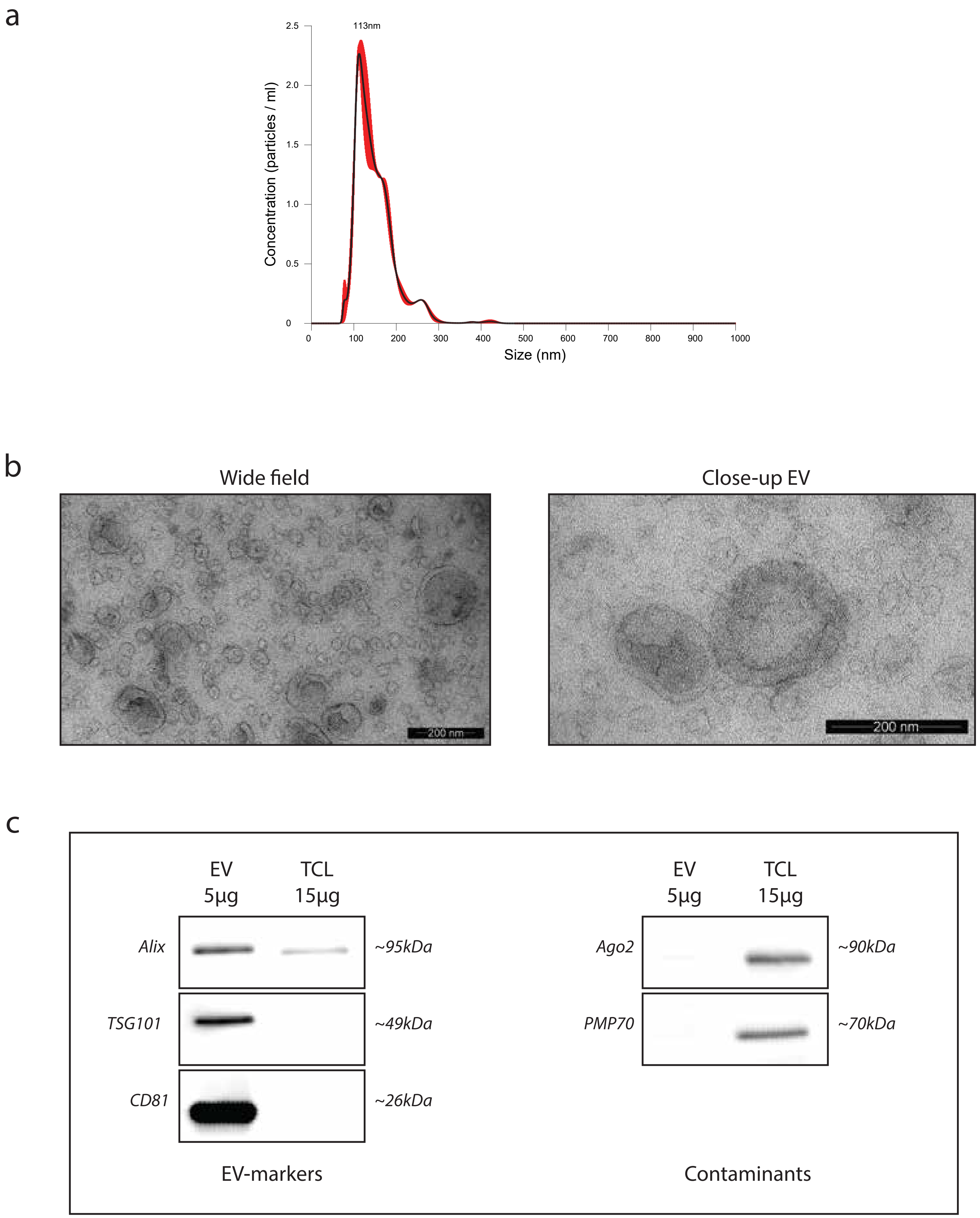

Supplementary figure 3: Characterization of MCF-7 Rab27b-GFP derived EVs
Implemented EVs were analyzed by (a) nanoparticle tracking analysis, (b) transmission electron microscopy and (c) Western blot analysis for EV-markers (Alix, TSG101, CD9, CD81) and contaminants (Ago2, GM130, Calreticulin, PMP70). 5µg of EV and 15µl of TCL were loaded. Original immunostaining results are shown in *Supplementary figure 10*.

Abbreviations:
GFP: green fluorescent protein. EV: extracellular vesicle. TCL: total cell lysate.

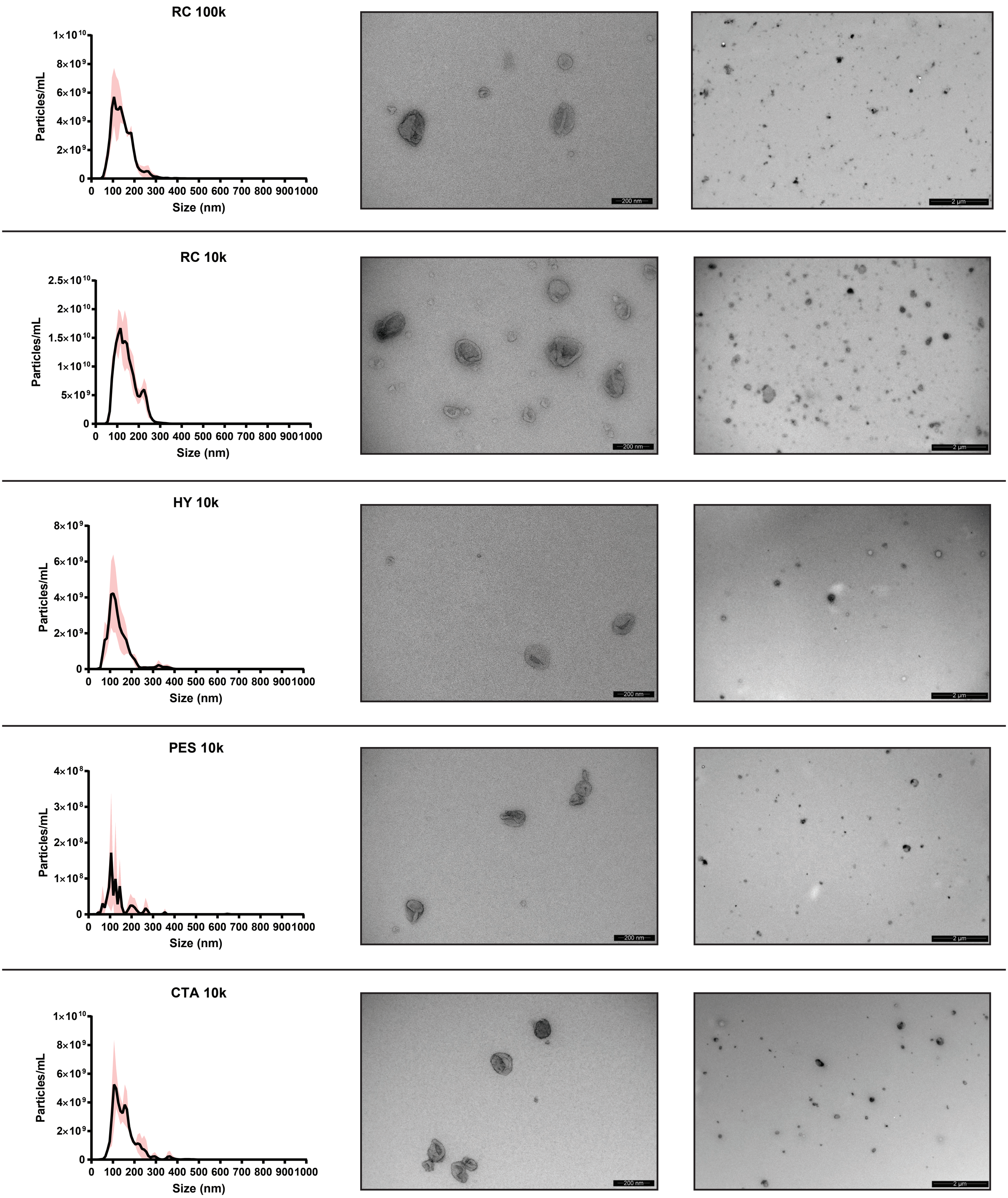

Supplementary figure 4: EV characterization after concentrating using centrifugal filter columns
Implemented EVs were analyzed by nanoparticle tracking analysis (left panel) and transmission electron microscopy (middle panel = close image, right panel = wide field image). NTA results are represented as mean (red area indicates standard deviation) of analysis from three replicate columns, where three videos of 60 seconds were recorded per column.

Abbreviations:
EV: extracellular vesicle. NTA: nanoparticle tracking analysis. RC: regenerated cellulose. HY: Hydrosart. PES: polyethersulfone. CTA: cellulose triacetate.

a

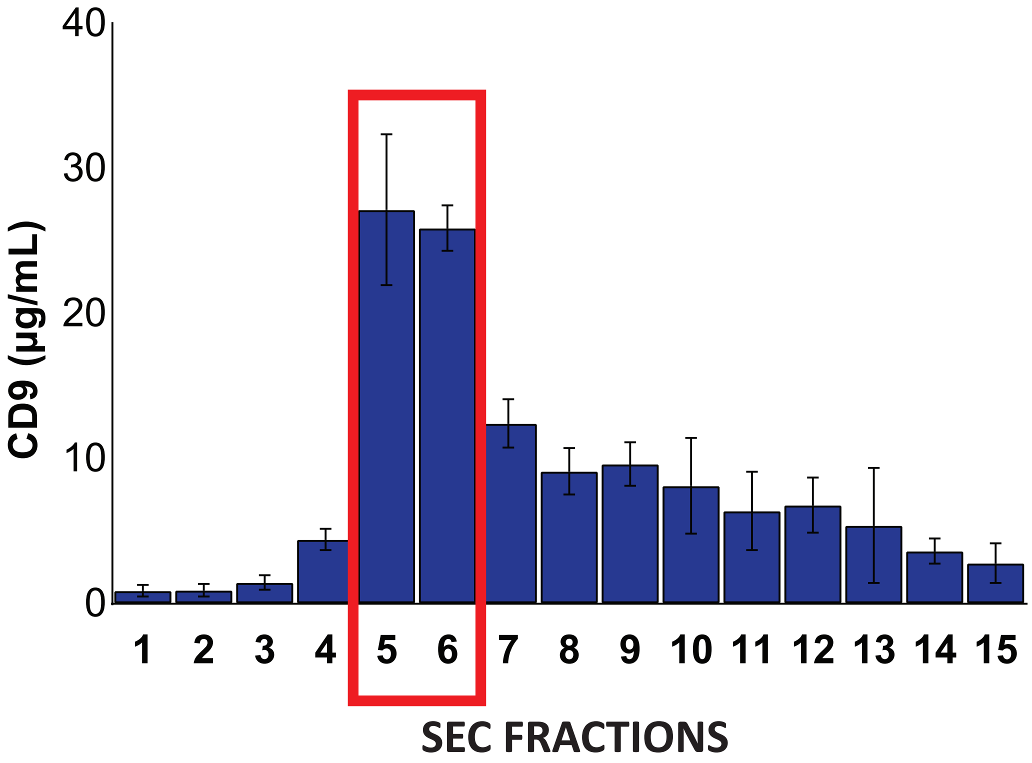

b

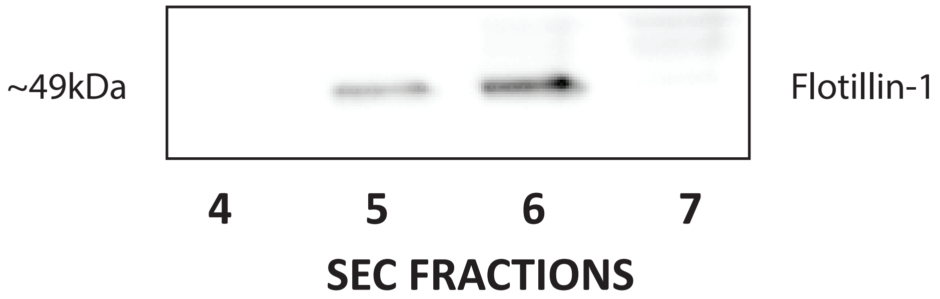

Supplementary figure 5: Preparation of plasma samples for centrifugal filters by SEC
Plasma (2mL) was loaded on top of a SEC column and 1mL elution fractions were collected. (a) ELISA for CD9 was performed on all consecutive SEC fractions. (b) Western blot analysis for Flotillin-1 was performed on the SEC fractions with the highest CD9 signal on ELISA, together with adjacent SEC fractions.
Mean values ± SD are indicated. Original immunostaining results are shown in *Supplementary figure 10*.

Abbreviations:
SEC: size-exclusion chromatography

a

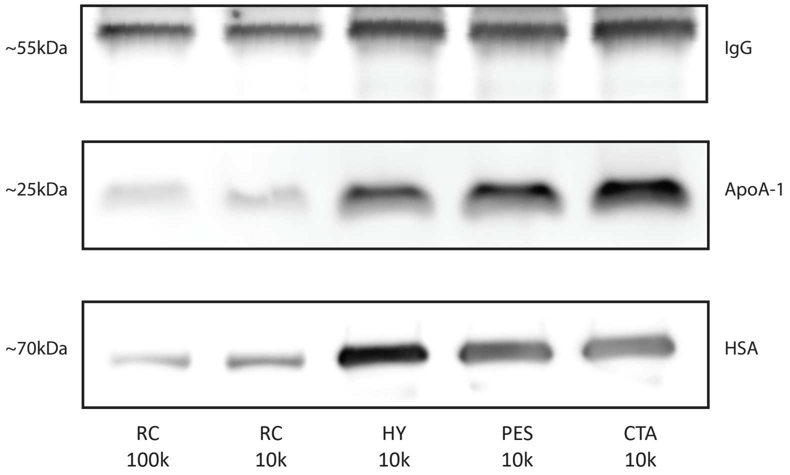

b

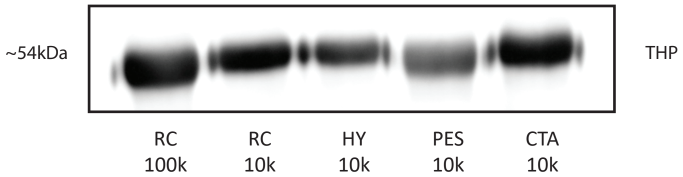

Supplementary figure 6: Analysis of centrifugal filter membrane lysates
Lysates of the centrifugal filter membranes were analysed by Western blot for adherence of soluble proteins and lipoproteins on the membranes after centrifugating using plasma and urine as sample. (a) For plasma samples human serum albumin, IgG and apolipoprotein A-1 were assessed. 15µg sample was loaded for each condition. (b) For urine samples Tamm-Horsfall protein was investigated. 7µg sample was loaded for each condition. Original immunostaining results are shown in *Supplementary figure 10*.

Abbreviations:
RC: regenerated cellulose. HY: Hydrosart. PES: polyethersulfone. CTA: cellulose triacetate. THP: Tamm-Horsfall protein. ApoA-1: apolipoprotein A-1. IgG: immunoglobulin G. HSA: human serum albumin.

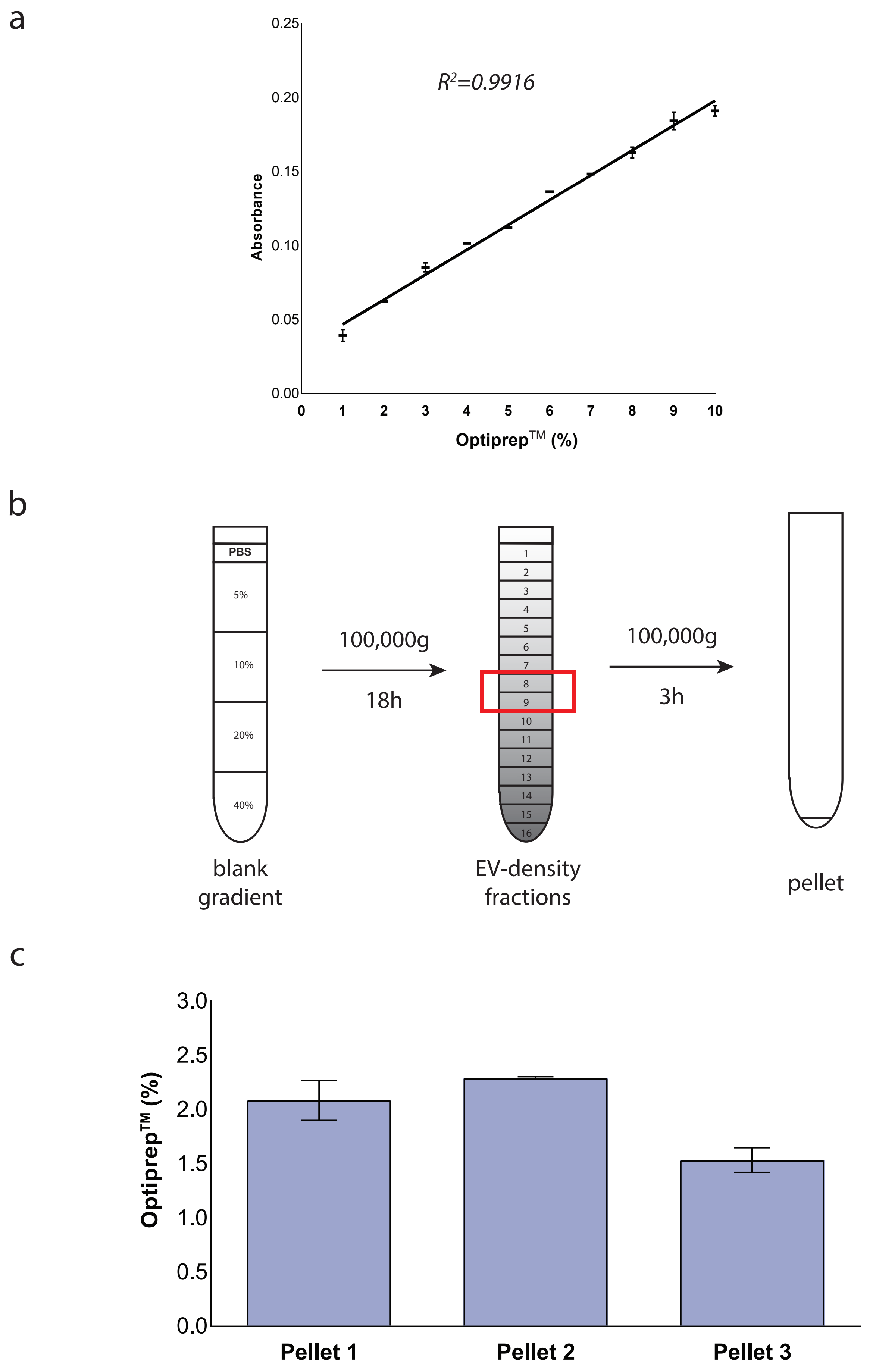

Supplementary figure 7: Quantification of Optiprep™ remnants using DC™ protein assay
(a) Serial dilutions of Optiprep™ were used to generate a standard curve with the DC™ protein assay according to manufacturer's instructions. Measured absorbance values are depicted for each dilution of Optiprep™. (b) Optiprep™ quantification was performed on three pelleted EV-corresponding density fractions of a blank density gradient. (c) Quantified Optiprep™ remnants in three independent pellets.
Mean values ± SD are indicated.

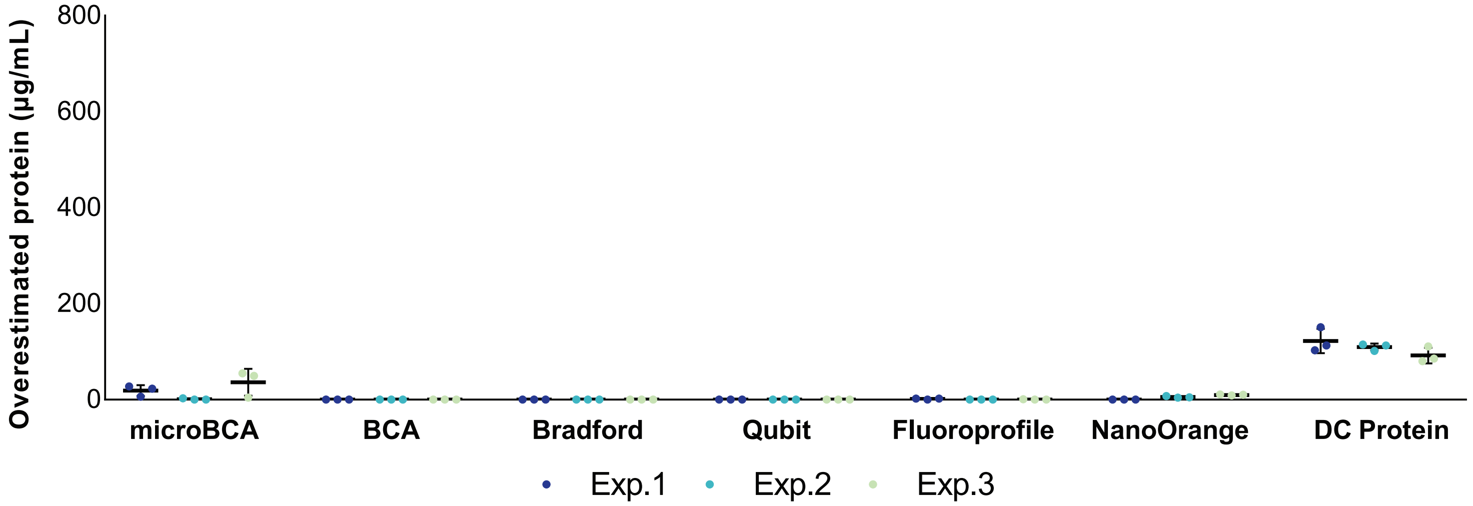

Supplementary figure 8: Intererence of Optiprep on protein assay kits
5µl PBS sample containing 3% Optiprep was used to determine interference of Optiprep on all implemented protein assay kits. Results are shown as resulting protein quantity determined by each protein assay kit. Three independent experiments were performed with each three replicates.

a

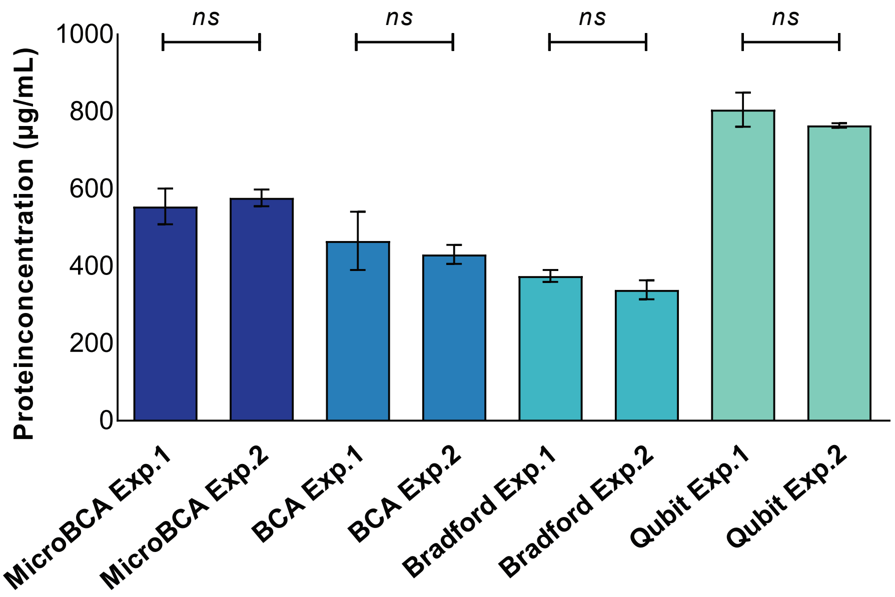

b

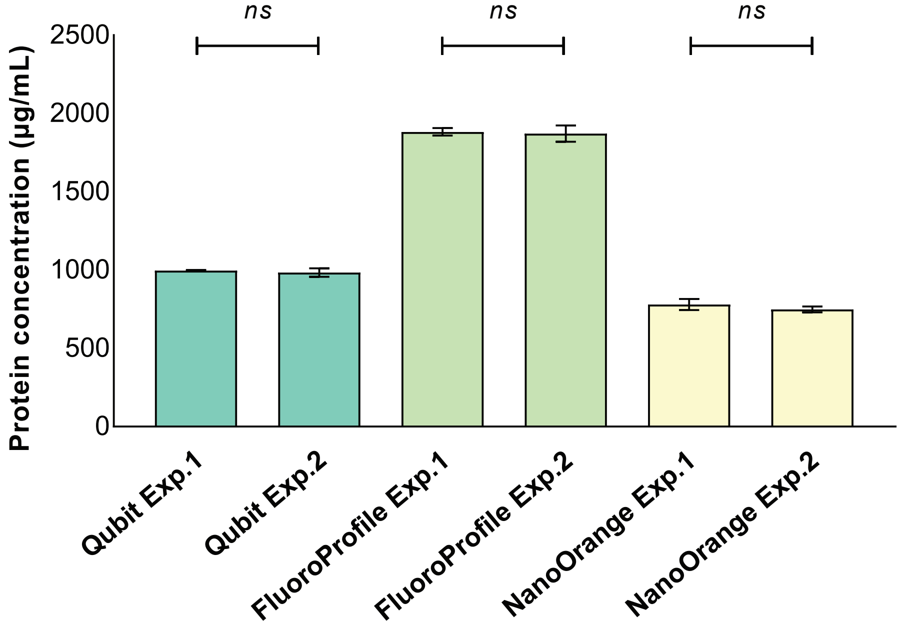

Supplementary figure 9: Influence of freeze-thawing on protein concentration measurements of EV samples

EV samples were measured before (Exp.1) and after (Exp.2) a freeze-thaw cycle at -80°C. (a) EV sample measured using three colorimetric (MicroBCA, BCA, Bradford) and one fluorometric (Qubit) assay. (b) Another EV sample measured using three fluorometric assays (Qubit, FluoroProfile, NanoOrange). Mean values $\pm$ SD are indicated. Significant differences were calculated using unpaired *t*-test (n=3, $p<0.5$).

Abbreviations:
ns = not significant

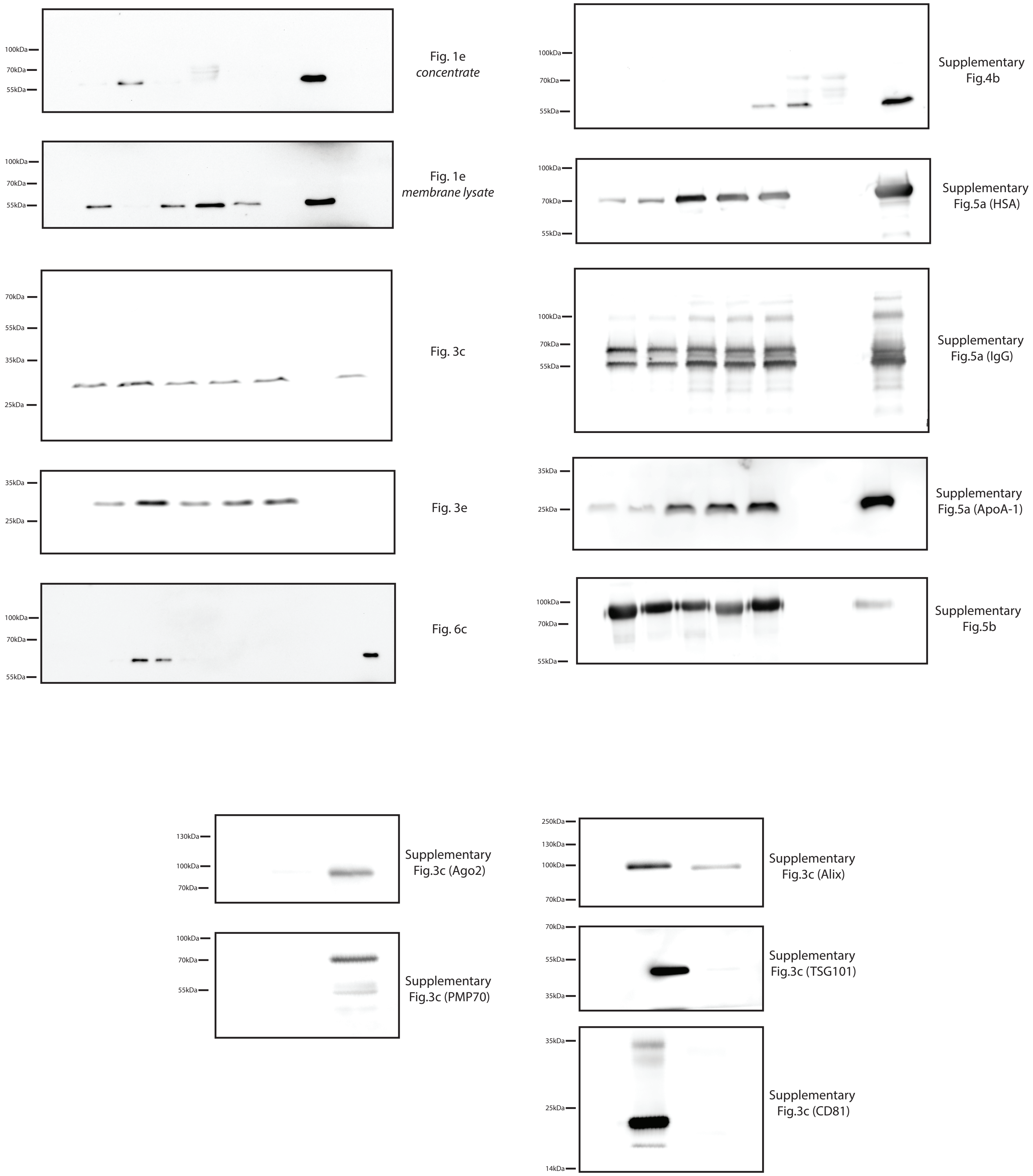

Supplementary figure 10: Original Western blot immunostaining results

**NOP10**	forward primer	GAAACGCTTCAAGGTGCTCAT
	reverse primer	GGTGGCAGAAAAGACATCAGTTTA
**OST4**	forward primer	ACGTGCAGCTCGCCATCT
	reverse primer	TGTTGACGGCCACGTAGTGA
**SNRPG**	forward primer	AGCGGGAGCGTGAGGAA
	reverse primer	TCGGGAGGGTGAGCTTTG
**TOMM7**	forward primer	GATTCCCGACGCTGTGGTT
	reverse primer	TCCCCTTGAAGAGCTGCTGTA

Supplementary table 1: Forward en reverse primers of assessed genes for RT-qPCR

		BCA	microBCA	Bradford	Qubit	FluoroProfile	NanoOrange	DC Protein
Assay principle		colorimetric	colorimetric	colorimetric	fluorometric	fluorometric	fluorometric	colorimetric
Incubation	Time	30min	120min	10min	15min	30min	10min	15min
	Temperature	37°C	37°C	RT	RT	RT	90°C	RT
Cost per sample*	Test tube	0,63 €	0,65 €	0,39 €	0,56 €	1,85 €	2,17 €	0,48 €
	Microplate	0,06 €	0,10 €	0,08 €	-	0,18 €	0,22 €	0,02 €
5µl sample	Used protocol	Microplate protocol	Microplate protocol	Micro microplate protocol	Standard protocol	Microplate protocol	Microplate protocol	Microplate protocol
	Dilution	5x	30x	30x	1x	10x	50x	1x
Apparatus		Plate reader or spectrophotometer	Plate reader or spectrophotometer	Plate reader or spectrophotometer	Qubit® fluorometer	Plate reader or fluorometer	Plate reader or fluorometer	Plate reader or spectrophotometer

Supplementary table 2: Comparison of seven different protein assays
General characteristics are summarized for four colorimetric (BCA, microBCA, Bradford, DC Protein) and three fluorometric (Qubit, FluoroProfile, NanoOrange) protein assays.

Abbreviations:
RT: room temperature.
